# Uronic acid metabolic process–related gene expression–based signature predicts overall survival of glioma

**DOI:** 10.1042/BSR20203051

**Published:** 2021-01-07

**Authors:** Yuemei Feng, Guanzhang Li, Zhongfang Shi, Xu Yan, Renpeng Li, You Zhai, Yuanhao Chang, Di Wang, Ulf Dietrich Kahlert, Wei Zhang, Fang Yuan

**Affiliations:** 1 Department of Pathophysiology, Beijing Neurosurgical Institute, Capital Medical University, China; 2 Department of Molecular Neuropathology, Beijing Neurosurgical Institute, Capital Medical University, China; 3 Department of Neurosurgery, Beijing Tiantan Hospital, Capital Medical University, China; 4 Neurosurgical Clinic, Medical Faculty, Heinrich-Heine University Duesseldorf, Germany; 5 Clinical Cooperation Unit Neuropathology, German Cancer Consortium (DKTK), Duesseldorf, Germany; 6 Clinical Center for Glioma, Capital Medical University, China; 7 Chinese Glioma Genome Atlas Network (CGGA) and Asian Glioma Genome Atlas Network (AGGA), China

**Keywords:** DNA replication, glioma, pentose phosphate pathway, prognosis, uronic acid metabolism

## Abstract

Glioma is the most common and malignant cancer of the central nervous system, and the prognosis is poor. Metabolic reprogramming is a common phenomenon that plays an important role in tumor progression including gliomas. Searching the representative process among numerous metabolic processes to evaluate the prognosis aside from the glycolytic pathway may be of great significance. A novel prediction signature was constructed in the present study based on gene expression. A total of 1027 glioma samples with clinical and RNA-seq data were used in the present study. Lasso-Cox, gene set variation analysis, Kaplan–Meier survival curve analysis, Cox regression, receiver operating characteristic curve, and elastic net were performed for constructing and verifying predictive models. The R programming language was used as the main tool for statistical analysis and graphical work. This signature was found to be stable in prognostic prediction in the Chinese Glioma Genome Atlas Network and the Cancer Genome Atlas databases. The possible mechanism was also explored, revealing that the aforementioned signature was closely related to DNA replication and ATP binding. In summary, a prognosis prediction signature for patients with glioma based on five genes was constructed and showed great potential for clinical application.

## Introduction

Glioma is a devastating malignant tumor in the central nervous system. The prognosis remains poor despite adopting aggressive treatment approaches, including neurosurgical resection followed by adjuvant chemo- and radiotherapy [1]. Therefore, the screening of the possible influencing factors for poor survival is of great significance for guiding clinical treatment.

The metabolic process plays an important role by providing metabolites and energy in both normal and cancer cell proliferation. Meanwhile, metabolic reprogramming is thought to be a new hallmark of cancer. Cancer cells, including glioma, have been reported to rely much more on glycolysis than on oxidative phosphorylation (OXPHOS) for energy metabolism, which is known as the ´Warburg effect’ [2]. Many other changes take place in cancer cells to adapt to new growth needs. For instance, the proportion of the pentose phosphate pathway (PPP) increases in colorectal cancer and negatively correlates with the prognosis of patients [3]. Higher fatty acid oxidation was found in patients with breast cancer [4] and even patients with glioma having a shorter survival [5]. Recent studies on altered metabolism and survival in glioma mainly focused on glycolysis metabolism [6–8], amino acid metabolism [9,10], and OXPHOS [11–13]. It is undeniable that other metabolic methods also play an important role in cancer cell proliferation. Therefore, which metabolic processes have an important impact on tumor progression and prognosis in glioma is a new research hotspot.

**Figure 1:**
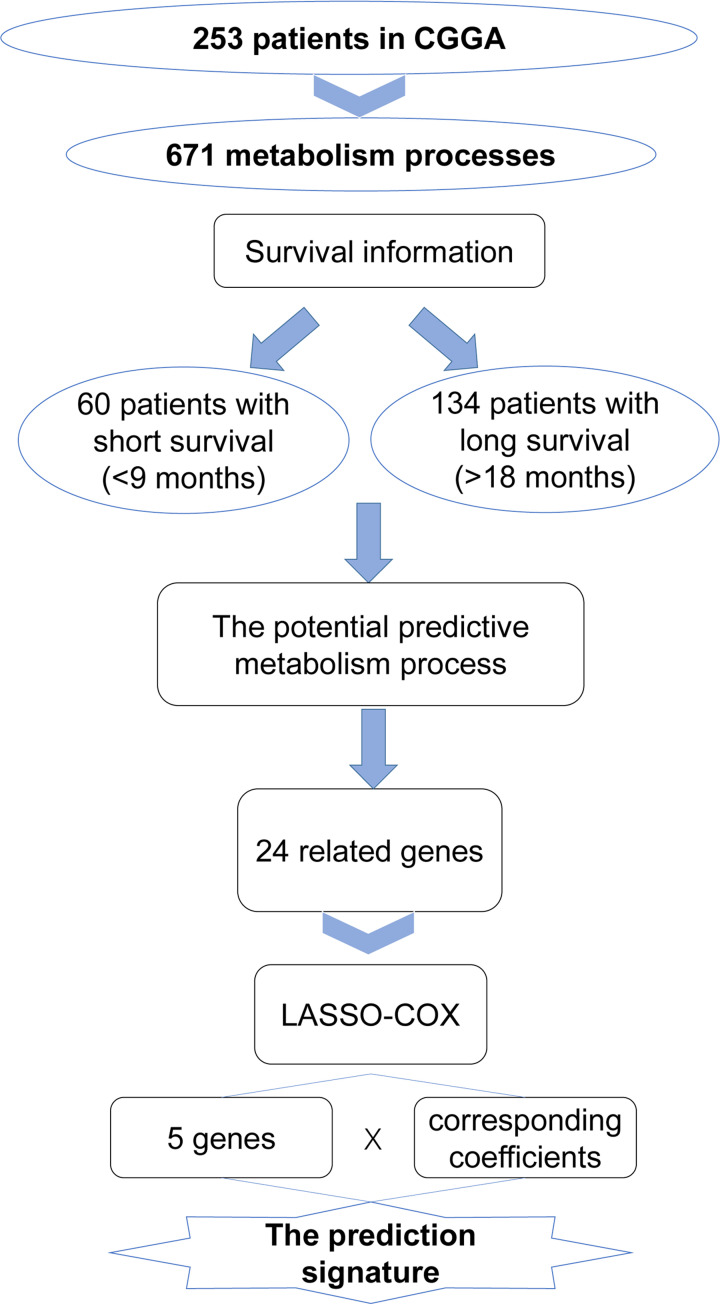
Workflow

In the present study, a rarely mentioned metabolic process was discovered to predict the overall survival (OS) of patients with glioma, and accordingly a gene signature was constructed. First, 671 metabolic processes were compared in CGGA and TCGA databases, and the uronic acid metabolic process was the most significant. Subsequently, genes related to the uronic acid metabolic process were screened by Lasso-Cox analysis [14]. Finally, five candidate genes were obtained to build a novel signature (Figure 1). The performance of the signature was then validated on the CGGA and TCGA databases, and its related biological functions were explored. The signature was found to be closely related to the DNA damage repair function and could be used to predict the prognosis of patients with glioma.

## Methods

### Patients and expression data processing

The present study involved data from 1027 patients with glioma sourced from the CGGA and TCGA RNA-seq databases. The clinical information is listed in Table 1. All the TCGA expression data and survival information were downloaded from the official TCGA website (https://cancergenome.nih.gov). Additionally, 253 patients with metabolic process–related data from the CGGA database were included in the present study. Each patient was diagnosed by two neuropathologists based on the 2007 World Health Organization (WHO) classification guidelines. Tumor samples were acquired from newly excised tissues. The OS was calculated from the date of diagnosis to the end of the follow-up investigation. Sample collection and data analyses were approved by the Beijing Tiantan Hospital institutional review board, and written informed consent was obtained from each participant. The study was conducted in accordance with the European Good Clinical Practice requirements (Declaration of Helsinki). The informed consent was obtained from all participants.

**Table 1:** Clinical information of patients

Characteristic	No. of patients (CGGA)	No. of patients (TCGA)
***Age at diagnosis***		
Mean	42.94	47.32
Standard deviation	11.93	15.29
***Gender***		
Male	203	354
Female	122	255
Not available	0	93
***Grade***		
WHO II	103	216
WHO III	79	241
WHO IV	139	152
Not available	4	93
***IDH1 Mutation***		
Mutation	175	428
Wildtype	149	234
Not available	1	40
***1p/19qCodeletionstatus***		
Codeletion	67	169
Non-codeletion	250	495
Not available	8	38

### Candidate risk signature building

Data from the CGGA database were identified as the experimental group, and those from the TCGA database was identified as the verification group. Based on previous findings, an OS time of fewer than 9 months was defined as short survival, and that more than 18 months was defined as long survival [15]. The Student’s *t* test was employed to analyze the data from the CGGA database. One metabolic process significantly associated with long or short survival (*P*<0.001) was obtained for subsequent analysis. After checking through the Gene Set Enrichment Analysis (GSEA) website (http://www.broadinstitute.org/gsea/), 24 process-related genes matched the CGGA database. Lasso-Cox dimension reduction analysis was performed via the *glmnet* and *survival* packages in R using the expression data of 24 genes and the survival information to get the most meaningful predictors. Finally, five genes and corresponding lambda values [UDP-glucuronic transferase (*UGT*)*8*: –0.031008518; dicarbonyl and L-xylulose reductase (*DCXR*): –0.039569338; sorbitol dehydrogenase (*SORD*): –0.081392798; xylulokinase (*XYLB*): 0.168664839; and crystallin lambda (*CRYL*) 1: –0.03481888] were obtained. The risk score of each patient was calculated from the sum of the corresponding gene values multiplied by the Lasso-Cox coefficient using the following formula: Risk score = (expr_gene1_ × coefficient _gene1_) + (expr_gene2_ × coefficient _gene2_) + … + (expr_genen_ × coefficient _genen_). The cutoff values of the risk groups were the median values of the risk scores in both the experimental and verification groups [16].

### Verification of the prediction model

The receiver operating characteristic (ROC) curve, the C-Index, and the calibration curve were employed in the analysis to verify the accuracy and specificity of the signature. An elastic net was used to ensure that the model was not overfitting.

### Biological functional enrichment scores

The biological functional enrichment score of each patient was generated via gene set variation analysis (GSVA) based on tumor transcriptome sequencing data. GSVA was performed using the default parameters of the GSVA package in R as described in a previous study [17]. The gene list for each biological function was downloaded from the AmiGO2 web portal (http://amigo.geneontology.org).

### Immunohistochemistry

Paraffin-embedded tissues with complete clinical information were obtained from the CGGA Tissue Bank. The present study was approved by the institutional review board (Number: KY-2020-093-02), and written informed consent was obtained from each patient. First, 4-µm sections were cut from paraffin-embedded tissues. The samples were deparaffinized in an oven at 65°C for 2 h. Then, they were rehydrated in xylene and decreasing concentrations of alcohol and washed with phosphate-buffered saline twice. After rehydration, antigen retrieval was performed with boiled Tris-EDTA buffer, pH 9.0. Immunohistochemical (IHC) analysis with the UGT8 antibody (Proteintech, WUHAN, China, 17982-1-AP, 1:100) was conducted according to the standard procedures. Images were taken with an optical microscope. The IHC results were assigned a mean score considering the multiplying of the intensity of staining and the proportion of the positive cells. Each section was independently assessed by two pathologists without prior knowledge of patient data. The intensity was scored as follows: 0, negative; 1, weak; 2, moderate; 3, strong. The frequency of positive cells was ranging from 0 to 100%. Positive reactions were defined as those showing brown signals. And the paraffin embedded human brain using 17982-1-AP at dilution of 1:100 was the positive control.

### Statistical analysis

All statistical analyses were conducted using the R programming language (https://www.r-project.org/, v3.5.0), SPSS 25.0 software, GraphPad Prism 7 software (GraphPad Software, Inc., CA, U.S.A.), and the Database for Annotation, Visualization and Integrated Discovery (DAVID) website (https://david.ncifcrf.gov/summary.jsp). Student’s *t* test and one-way analysis of variance were used to compare the gene expression levels, risk scores, and other factors among different subgroups. The prognostic significance was assessed using Kaplan–Meier curves employing the calculated risk score by the RNA-seq database and the survival information of patients. Kaplan–Meier survival curve analysis was carried out to illustrate the signature survival differences observed between high- and low-risk groups. The correlations between two variables were verified by Pearson’s correlation analysis, and *P*<0.05 indicated a statistically significant difference.

## Results

### A new metabolic process related to glioma prognosis and a novel signature built based on five genes

**Figure 2:**
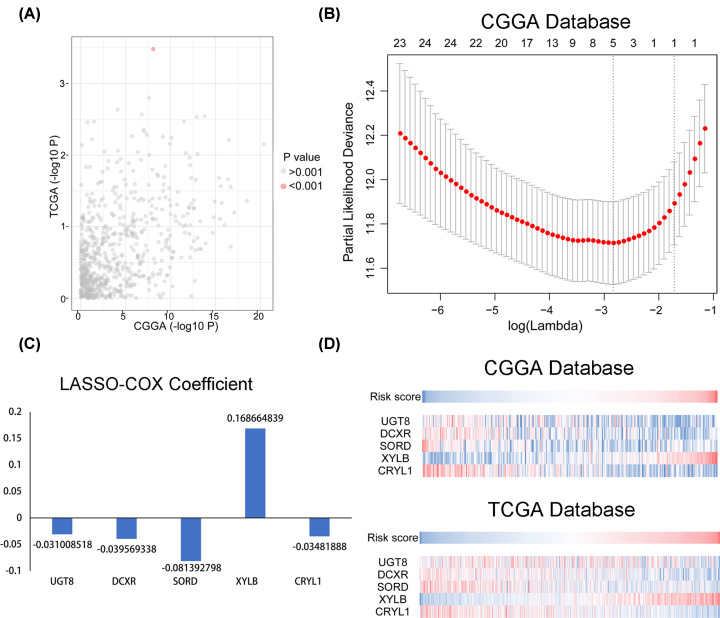
Analysis of metabolic processes using the CGGA database (**A**) Different metabolic processes between long and short survival. (**B**) Differentially expressed genes determined by Lasso-Cox. (**C**) Coefficients of five genes selected by Lasso-Cox. (**D**) A novel risk score calculated based on coefficients and the CGGA database and validated in the TCGA database.

**Figure 3:**
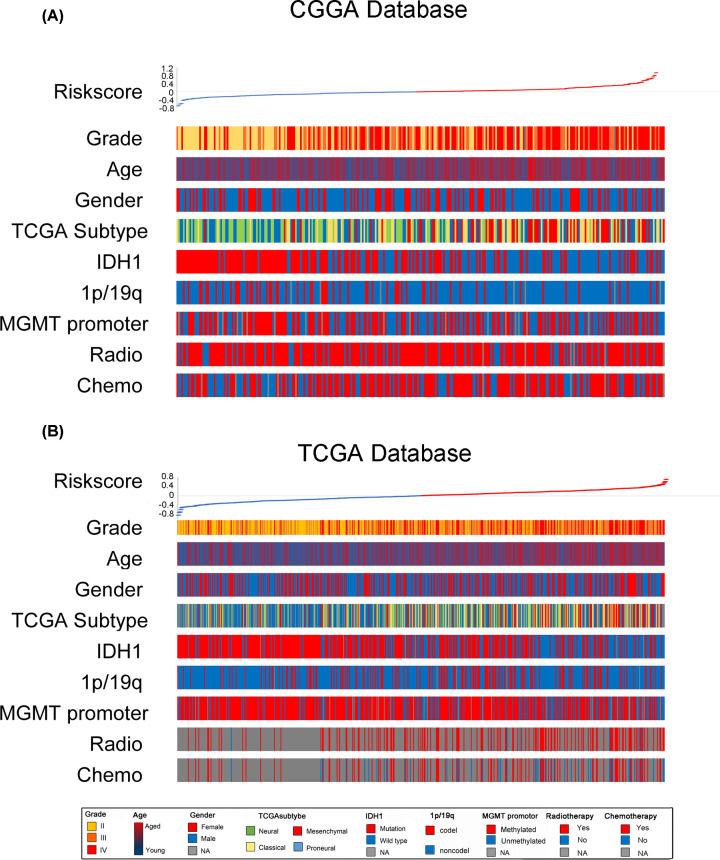
Relationships between risk score and clinical information (**A**) in the CGGA database and (**B**) in the TCGA database.

A total of 671 metabolic processes were identified. A *t* test was performed to filter the metabolic processes most related to OS in both the CGGA and TCGA databases. The result showed that the uronic acid metabolic process stood out (*P*<0.001; Figure 2A). In the training group, the CGGA database was used to search for related genes. Ultimately, 24 genes were found to contribute to the uronic acid metabolic process. Five probes and their corresponding coefficients were identified by Lasso-Cox dimension reduction analysis to screen out further the most representative genes (Figure 2B,C). Finally, a novel prognostic and uronic acid metabolic process capability signature was constructed based on the five genes (Figure 2D). The risk signature was summarized using the following formula: Risk score = (–0.031008518 × Expr _UGT8_) + (–0.039569338 × Expr _DCXR_) + (–0.081392798 × Expr _SORD_) + (0.168664839 × Expr _XYLB_) + (–0.03481888 × Expr _CRYL1_). The risk score distribution of each sample in the CGGA and TCGA groups were displayed in the scatter diagrams (Figure 3A,B). The in-depth analysis of this prediction signature in gliomas was conducted as follows.

### Landscape of signature and clinical characteristics in gliomas

The prediction signature was applied to the CGGA database to validate its predictive performance further. The risk scores of 325 patients in the CGGA database were calculated based on gene expression, and the median of the score was defined as the cutoff. A heatmap revealed the relationships between risk scores and WHO grade, age, sex, TCGA subtype, IDH1 status, 1p/19q status, O-6-methylguanine-DNA methyltransferase (MGMT) promoter, radiotherapy, and chemotherapy status (Figure 3A). It was determined that, excluding sex, each characteristic had an asymmetrical distribution. Higher grade, older aged, IDH1-wild, 1p/19q-non-codeletion, and unmethylated-MGMT promoter patients were mostly distributed in the higher-risk segment. The same method was used to investigate the TCGA database, and 702 patients were included (Figure 3B). The relationships between the risk scores and WHO grade, age, IDH1, and 1p/19q status in the TCGA database were the same as those in the CGGA database. The statistical analysis of these relationships was subsequently carried out.

### Relationship between signature and clinical characteristics in gliomas

**Figure 4:**
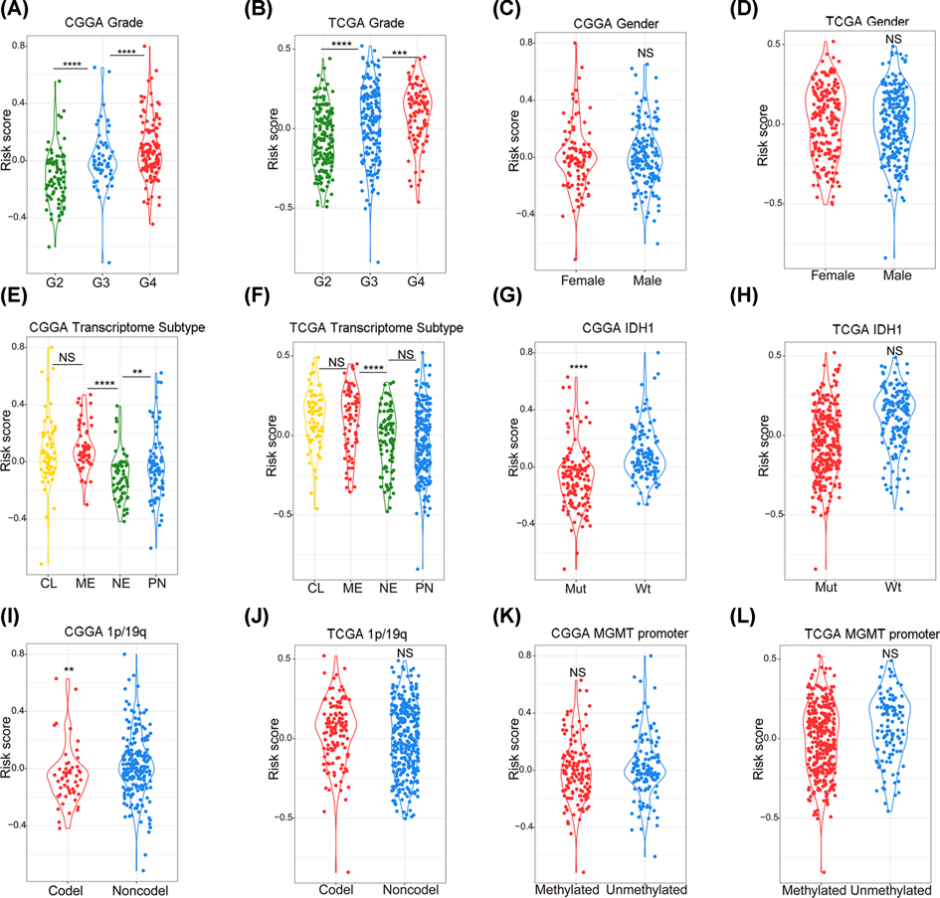
Correlations between risk scores and clinical features in the CGGA and TCGA databases (**A** and **B**) Correlation between risk score and WHO grade in the CGGA and TCGA databases. (**C** and **D**) Correlation between risk score and sex in the CGGA and TCGA databases. (**E** and **F**) Correlation between risk score and transcriptome subtype in the CGGA and TCGA databases. (**G** and** H**) Correlation between risk score and IDH1 status in the CGGA and TCGA databases. (**I** and **J**) Correlation between risk score and 1p/19q status in the CGGA and TCGA databases. (**K** and **L**) Correlation between risk score and MGMT promoter status in the CGGA and TCGA databases.NS: *P*>0.05, **P<0.01, ***P<0.001, ****P<0.0001.

The relationships between the signature risk score and various clinical features were investigated via the CGGA and TCGA databases. In the CGGA database, it was found that the risk score was higher in WHO IV gliomas than in WHO II and WHO III gliomas (Figure 4A), and the same trend was found in the TCGA database (Figure 4B). In both the CGGA and TCGA databases, the risk scores between different sexes were found to have no significant differences (Figure 4C,D). Among the transcriptome subtypes, the risk score was found to be relatively higher in the mesenchymal subtype (Figure 4E,F). In terms of molecular pathology, the risk score was found to be much higher in IDH1 wild-type gliomas (Figure 4G,H). In 1p/19q-non-codeletion gliomas, a higher risk score was observed (Figure 4I,J). No correlation was found between the MGMT methylation status and the built signature (Figure 4K,L).

### Signature was closely related to DNA replication and ATP binding

**Figure 5:**
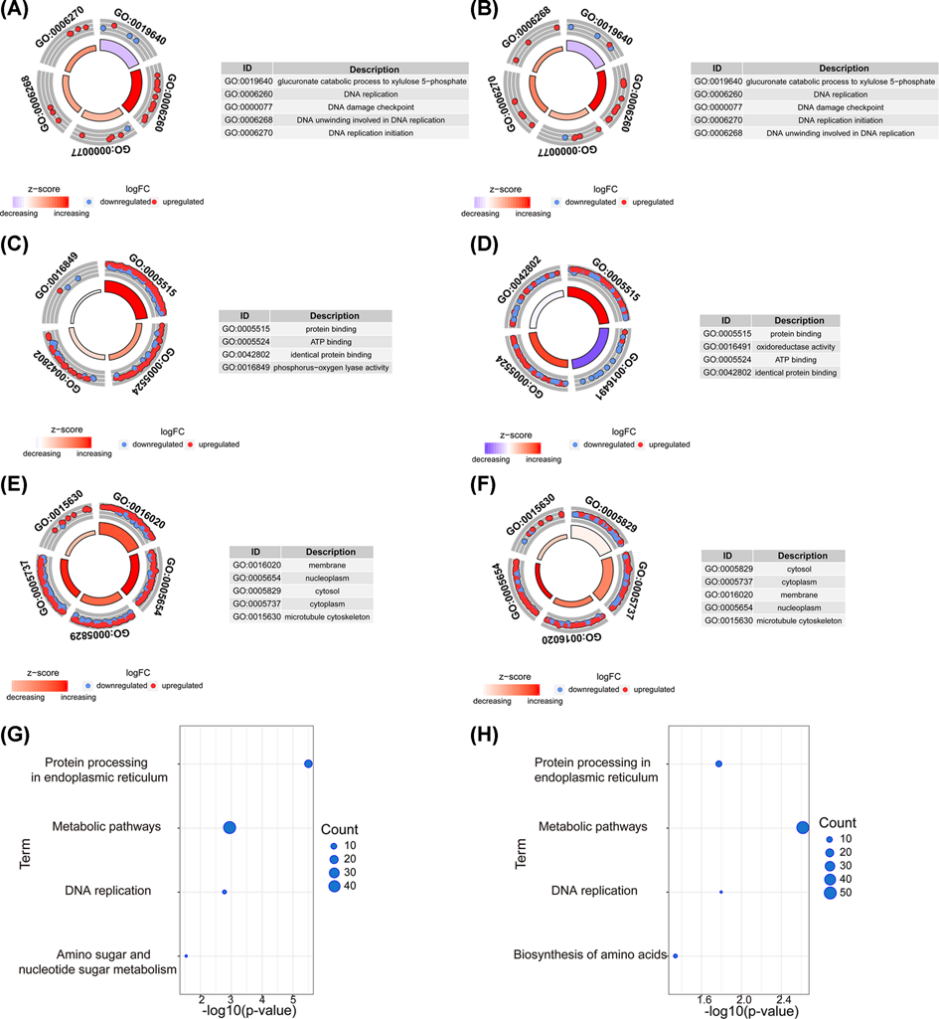
Risk score−related biological functions (**A** and **B**) Related biological processes in the CGGA and TCGA databases. (**C** and **D**) Related molecular functions in the CGGA and TCGA databases. (**E** and **F**) Related cellular components in the CGGA and TCGA databases. (**G** and **H**) Related KEGG pathways in the CGGA and TCGA databases.

Gene ontology function enrichment analysis was performed to explore the biological functions, including biological process, molecular function, and cellular component associated with the signature. ´Glucuronate catabolic process to xylulose 5-phosphate’, ´DNA replication’, ´DNA damage checkpoint’, ´DNA unwinding involved in DNA replication’, and ´DNA replication initiation’ were underlined as risk score–related biological processes in the CGGA database (Figure 5A). Consistently, the result in the TCGA database was obtained as a validation (Figure 5B). Risk score–related molecular functions focused more on ´ATP binding’ and ´protein binding’. (Figure 5C,D). They were more related to ´cytosol’ and ´nucleoplasm’ in the CGGA and TCGA databases (Figure 5E,F). Otherwise, the study explored the Kyoto Encyclopedia of Genes and Genomes (KEGG) pathway in both the CGGA and TCGA databases and found much more enrichment in ´protein processing in endoplasmic reticulum’ and ´metabolic pathways’, as well as in ´DNA replication’, ´amino sugar and nucleotide sugar metabolism’, or ´biosynthesis of amino acids’ in the CGGA and TCGA databases (Figure 5G,H).

### Signature could predict survival of patients with glioma independently

**Figure 6:**
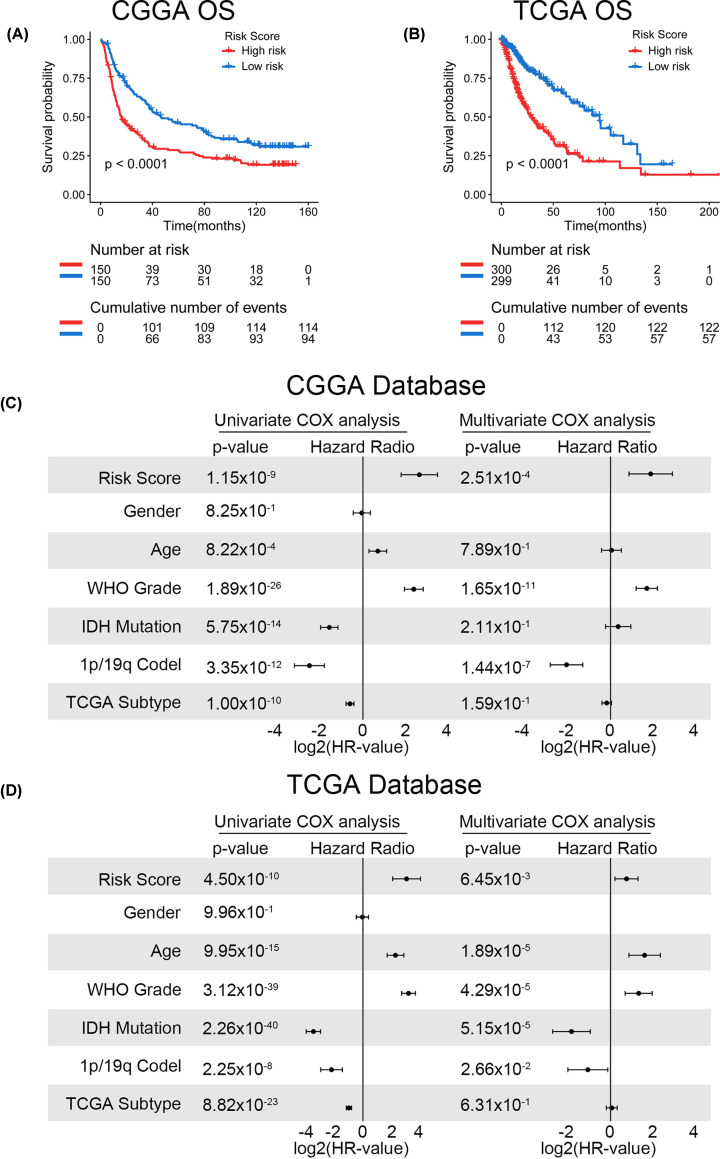
Signature was found to well predict the survival information (**A** and **B**) Risk score applied to survival information in the CGGA and TCGA databases (*P*<0.0001). (**C** and **D**) Univariate and multivariate Cox regression in the CGGA and TCGA databases.

**Figure 7:**
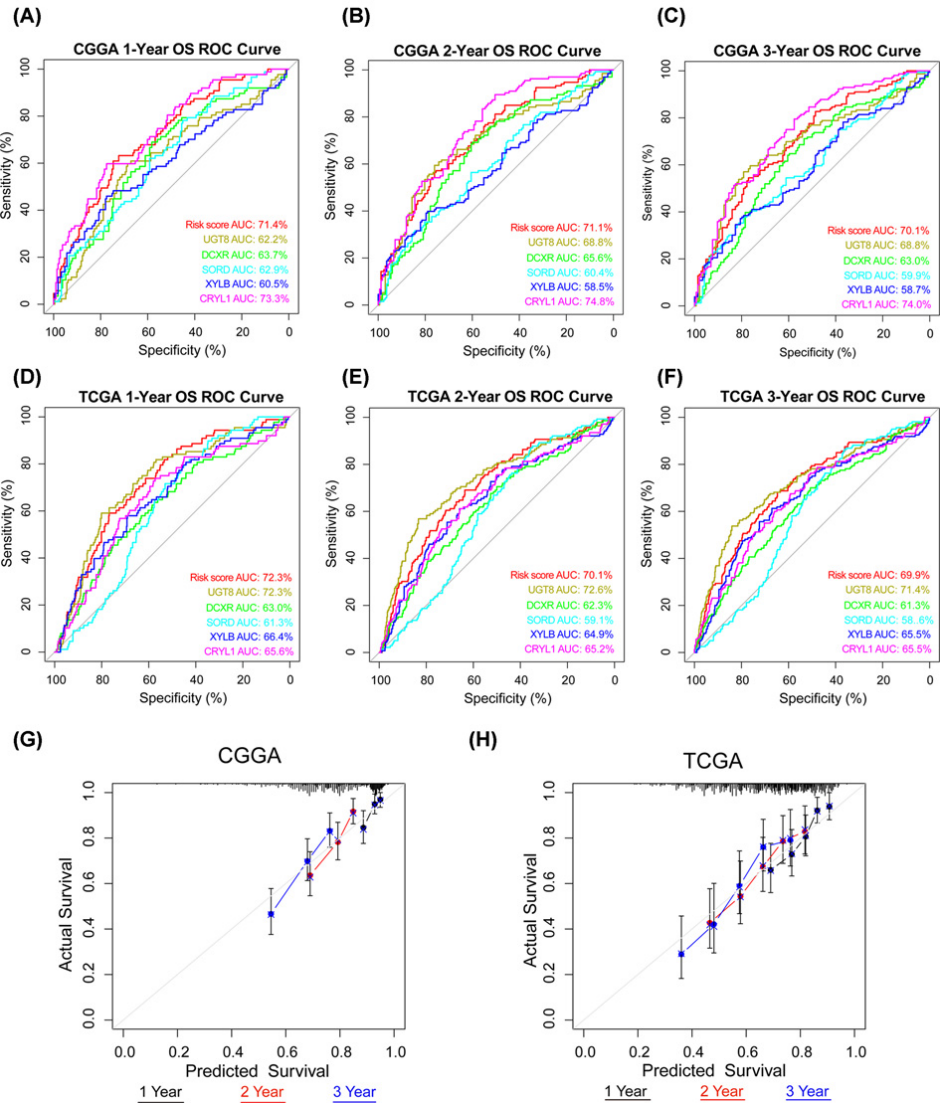
Accuracy of the risk prediction model (**A-C**) ROC curve of the signature in 1, 2, and 3 years in the CGGA database. (**D–F**) ROC curve of the signature in 1, 2, and 3 years in the TCGA database. (**G** and **H**) Calibration curve of the signature model in 1, 2, and 3 years in the CGGA and TCGA databases.

**Figure 8:**
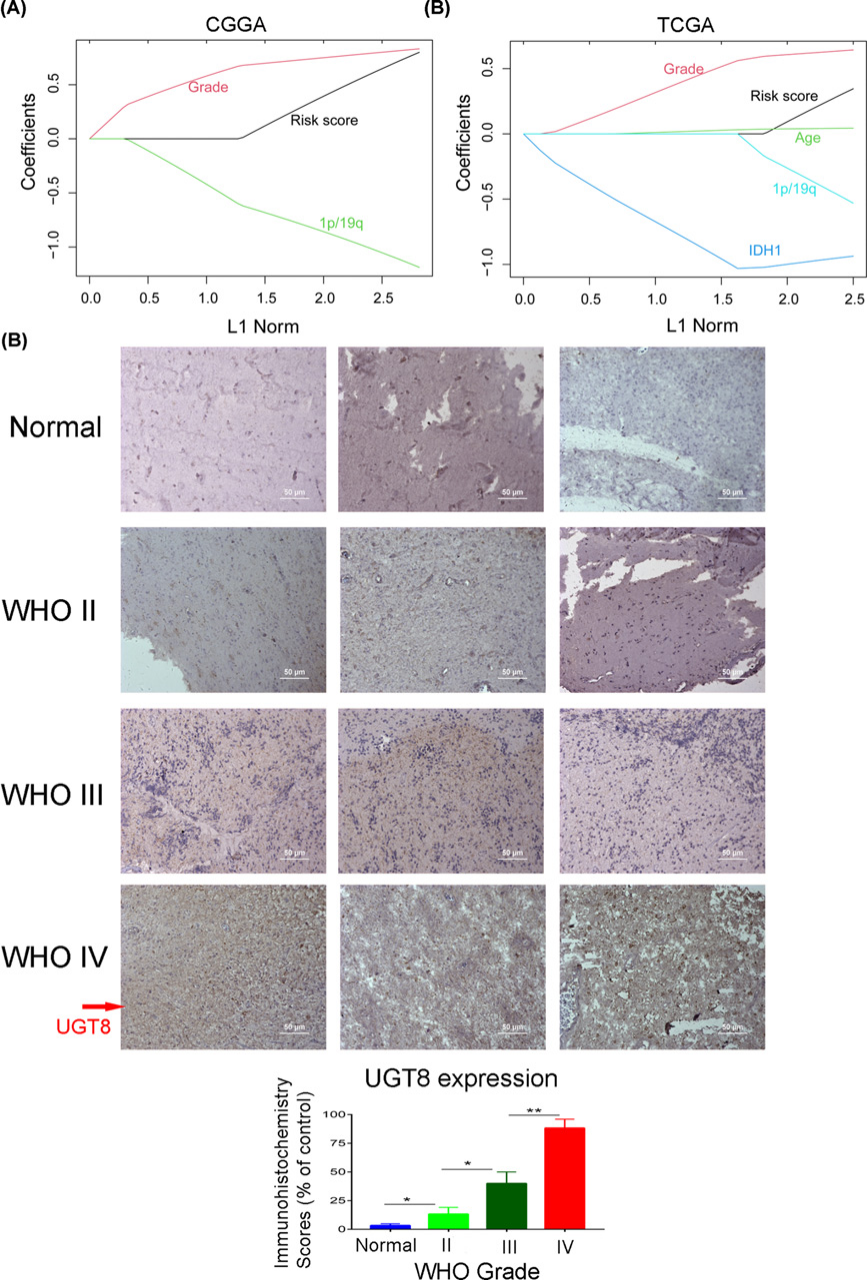
Accuracy of the risk prediction model (**A** and **B**) The elastic net in the CGGA and TCGA databases. (**C**) IHC of UGT8 in normal, WHO II, WHO III, and WHO IV, respectively (*n*=3, scale bar, 50 μm; *P<0.05, **P<0.01).

The signature was applied to the CGGA and TCGA databases to evaluate its performance in terms of prognostic prediction. As shown in Figure 6A,B, patients in the high-risk group had a shorter OS compared with those in the low-risk group in both the CGGA and TCGA databases (*P*<0.001). In addition, univariate and multivariate Cox regression analyses were performed to evaluate the comprehensive prognostic prediction value of the risk score and clinicopathological characteristics. Univariate Cox regression was employed in risk score, sex, age, WHO grade, IDH1 status, 1p/19q status, and TCGA subtype. The risk score, age, WHO grade, IDH1 status, 1p/19q status, and TCGA subtype were notably related to the survival in the CGGA database (*P*<0.001). These factors were included in multivariate Cox regression and revealed that the risk score, WHO grade, and 1p/19q status were screened as independent prognostic factors (*P*<0.05; Figure 6C). Consistently, in the TCGA database, the risk score, WHO grade, IDH1 status, and 1p/19q status were found to be independent prognostic factors (Figure 6D). In summary, the results showed that the built signature could predict the survival of patients with glioma as an independent factor. The ROC curve showed that the risk score has better efficiency compared with the five genes predicting the 1-, 2-, and 3-year survival (Figure 7A–F). Otherwise, the calibration curve was displayed and proved that the risk score had a high accuracy of the model in 3 years, and the value of the C-Index was 0.641 and 0.652 in the CGGA and TCGA databases, respectively (Figure 7G,H). As shown in Figure 8A, the risk score was better fitting than grade and 1p/19q status. Consistently, the risk score was better fitting than grade, age, IDH1 status, and 1p/19q status (Figure 8B). The bioinformatics analysis results were verified through IHC. The results showed that the expression of UGT8 increased in patients with the WHO grade (Figure 8C).

## Discussion

The metabolism in malignant gliomas is an evolving field. Previous findings indicate that altering metabolic processes played important roles in glioma progression [18]. These adjustments of the proportion of different metabolic pathways led to the growth of glioma by obtaining sufficient materials for biosynthesis. Typically, the glycolysis metabolism increased despite sufficient oxygen in many tumors, a phenomenon known as the ‘Warburg effect’ [18]. Meanwhile, these adjustments were also beneficial to resistance to chemotherapy and radiotherapy [19].

The present study was novel in building a uronic acid metabolic process–related signature involving five genes (*UGT8, DCXR, SORD, XYLB*, and *CRYL1*) to predict the survival of glioma in the CGGA database. The uronic acid metabolism is another sugar catabolism pathway to provide xylulose 5-phosphate for the PPP, accounting for only a small part of glucose metabolism. The PPP was obviously up-regulated in glioma, contributing to tumor proliferation and progression, and impacted the treatment effect finally [20].

The subsequent analysis verified that the signature worked as an independent prognostic factor in both the CGGA and TCGA databases. A low-score patient would have better survival than a high-score patient. Next, the study explored the related biological functions; finding the signature was related to DNA replication and ATP binding. The results of the KEGG pathway conveyed that ‘protein processing in endoplasmic reticulum’ and ‘metabolism pathways’ had a close relationship with the signature built in the present study. As known, the uronic acid metabolic process can produce uridine diphosphate glucose, which is related to glycogen synthesis, and uridine diphosphate glucuronic acid (UDPGA), which can produce xylulose 5-phosphate, react with nicotinamide adenine dinucleotide phosphate (NADPH) and enter the PPP [21]. Consistently, the UDPGA is catalyzed by UDP-glucuronic transferase (UGT) on the endoplasmic reticulum membrane to degrade drugs. It is hypothesized that the uronic acid metabolic process contributes to drug resistance in glioma.

The biological functions of the aforementioned five genes were reviewed to explore the mechanism of the risk score in prediction. The protein encoded by *UGT8* is a member of the UDP-glycosyltransferase family. It catalyzes the transfer of galactose to ceramide, a key enzymatic step in the biosynthesis of galactocerebrosides, which are abundant sphingolipids in the myelin membrane of the central and peripheral nervous systems. UGT8 has been reported to be related to poor prognosis in basal-like breast cancer [22]. However, inhibiting UGT8 alone could not inhibit cell proliferation and invasion of non-small cell lung carcinoma [23]. In the present study, the expression of *UGT8* was obviously related to the WHO grade. *DCXR* encodes the protein, which is a ‘moonlighting protein’ [24] and acts as a homotetramer to catalyze diacetyl reductase and L-xylulose reductase reactions, but a small proportion is expressed in the brain. The encoded protein may play a role in the uronate cycle of glucose metabolism and in cellular osmoregulation in the proximal renal tubules. Defects in this gene are a cause of pentosuria. Recent observations associate DCXR with a role in cell adhesion, pointing to a novel function involving tumor progression and possibly metastasis [24]. *SORD* is less expressed in the brain and encodes the sorbitol dehydrogenase catalyzing the interconversion of polyols and their corresponding ketoses, and, together with aldose reductase, making up the sorbitol pathway. *XYLB* encodes the protein belonging to a family of enzymes that include fucokinase, glucokinase, glycerokinase, and xylulose kinase. These proteins play important roles in energy metabolism. *CRYL1* is a well-known tumor suppressor in hepatocellular carcinoma [25], and is related to the uronate cycle functions as an alternative glucose metabolic pathway. However, the role of these five genes in glioma progression is not clear to date.

In conclusion, a signature related to uronic acid metabolism was built, which could predict the survival of patients with glioma. The relationships between risk score and glioma clinical features and molecular pathology were revealed on the macroscopic scale. The risk score was associated with DNA damage repair function. The retrospective studies revealed that the risk score could predict the prognosis of patients with glioma. These three aspects provided possible solutions to the clinical problem of judging the treatment effect of gliomas.

## Data Availability

The TCGA and CGGA data used to support the findings of this study were sourced from https://cancergenome.nih.gov/ and www.cgga.org.cn/, respectively (see Supplementary Material).

## Abbreviations

NADPH: nicotinamide adenine dinucleotide phosphate
OS: overall survival
OXPHOS: oxidative phosphorylation
PPP: pentose phosphate pathway
UDPGA: uridine diphosphate glucuronic acid
UGT: UDP-glucuronic transferase
